# Supplementary material for: Facilitators, barriers, and strategies for the implementation of peer-led tuberculosis active case finding among people who use drugs in Dar es Salaam, Tanzania
Source: PLoS One. 2025 May 28;20(5):e0310069. doi: 10.1371/journal.pone.0310069 (PMC12118968; doi:10.1371/journal.pone.0310069)
Supplement: S1 File — (PDF) [file pone.0310069.s001.pdf]

Tuberculosis Active Case Finding among People Who Use Drugs (PWUDs)

In-depth interview – Peer People who Use Drugs

(Version 1.2 dated 20 October 2022)

Interview Information:
Name of Interviewer:
Location:
Interviewee code:
Date:

Introductory Remarks:
Thank you for consenting to participate in this interview. I am......................... and I would like to discuss about your views in relation to Tuberculosis (TB) active case finding (ACF) among people who use drugs (PWUDs). The study is being done by the National Institute for Medical Research – Mbeya Medical Research Centre and Ifakara Health Institute in collaboration with the Swiss Tropical and Public Health Institute, Switzerland.

Closed Questions:
Participant Identification

a) Name of health facility - MAT clinic_______________________________________
b) Name of district: ____

c) Age (years): ____

d) Sex: ☐ Male ☐ Female

e) Peer category (select all that apply
   i. ☐ PWUD attending MAT clinic
   ii. ☐ Prior history of TB treatment
   iii. ☐ HIV client
   iv. ☐ Others (specify) ____

f) Level of education
   i. ☐ Primary education
   ii. ☐ Secondary education and above
   iii. ☐ Vocational training
   iv. ☐ Others

g) Occupation
   i. Employed (specify) ____
      - Formal
      - Informal
      - NGO supported
   ii. Unemployed

h) How many years have you been enrolled in the MAT clinic? (if applicable) [__|__]
i) How many years of experience in TB ACF? [__|__]

Open Questions:
Experience in TB ACF

1. What is your opinion about TB among PWUDs?
2. What do you understand when you hear about TB ACF in PWUDs?
3. Why are TB active case finding activities conducted among PWUDs? Advantages

4. I want to understand how you go about finding and screening PWUDs for the TB ACF program. Can you walk me through your process from start to finish?

- What tools/materials do you need as a peer e.g., screening tools, diagnostic tools?
    - How do you decide on where to go and who to screen?
    - Screening tools
    - Sample collection, dx and result feedback
    - Linkage to care
    - Close contact screening?
    - Frequency of TB ACF activities

5. How do you establish trust to gain entry into this population?
    - Rapport-building techniques, examples
    - Do you feel accepted by PWUDs / health worker?

6. Could you please tell me what makes it difficult for you to conduct TB ACF among PWUDs?
    - client refusal or uncooperative – why? with examples
    - frequent mobility of PWUDs
    - stigma surrounding PWUDs
    - fear of being caught by the police? run away?
    - violence
    - gender – influence in being a female or a male peer? why, with examples
    - lack of incentive / remuneration

7. How do you address / deal with the difficulties mentioned in question 6 above?

8. Could you please tell me about what helps you when conducting TB ACF?
    - financial support
    - facility support
    - community based organizations
    - peer influence
    - recommended changes for improvement

9. Basing on your interactions with HCW and CHW, how do PWUDs perceive the implementation of TB screening activities?
    - Consent
    - Fears
    - Refusal

10. What is your understanding on needle syringe exchange programs (NSP) and your opinion in using these programs in accessing PWUDs during TB screening and treatment?

11. As a peer PWUD, how were you selected to perform TB ACF?
    - random / selective selection, explain
    - training received – duration, frequency, last training
    - what is it like for you to do this work?
    - how do you feel being around other PWUDs in the community?

12. Do you have any additional comments on this topic that you would like to share?

End of interview
Thank the participant/respondent

Uibuaji wa Kifua Kikuu miongoni mwa Waraibu wa Dawa za Kulevya (PWUDs)

Mahojiano ya Kina – Kundi rika Waraibu wa Dawa za Kulevya

(Toleo la 1.2 tarehe 20 Octoba 2022)

Taarifa ya Mahojiano:
Jina la anaehoji:
Eneo:
Nambari ya anaehojiwa:
Tarehe:

Taarifa za Utangulizi:
Asante Kwa kukubali kushiriki kwenye mahojiano haya. Mimi ni ....na ningependa tujadili kuhusu maoni yako kuhusuiana na Uibuaji wa Kifua kikuu kwenye jamii kwa waraibu wa dawa za kulevya. Utafiti unafanywa na Taasisi ya Taifa ya Utafiti wa Magonjwa ya Binadamu - Kituo Cha Utafiti Mbeya na Taasisi ya Afya Ifakara Kwa kushirikiana na Taasisi ya Afya ya Jamii ya Swiss Tropical, Uswisi.

Maswali yaliyofungwa:
Utambulisho wa Mshiriki

a) Jina la kituo Cha Afya - Kliniki ya MAT____________________________________
b) Jina la Wilaya: ____

c) Umri (miaka): ____

d) Jinsia: ☐ Me ☐ Ke

e) Aina ya rika (chagua yote yanayohusika)
   i. ☐ Watu wanaotumia Dawa za Kulevya wanaohudhuria MAT clinic
   ii. ☐ Historia ya awali kabla ya matibabu ya TB
   iii. ☐ Mteja wa UKIMWI
   iv. ☐ Nyingine (taja) ____

f) Kiwango Cha elimu
   i. ☐ Elimu ya msingi
   ii. ☐ Elimu ya sekondari na kuendelea
   iii. ☐ Mafunzo ya ufundi
   iv. ☐ Nyingine

g) Kazi
   i. Ameajiriwa (taja) ____
      - Rasmi
      - Sio rasmi
      - Anasaidiwa na NGO
   ii. Hajaajiriwa

h) Je, umejiandikisha katika kliniki ya MAT kwa miaka mingapi? (Kama inahusika) [__|__]
i) Una uzoefu wa miaka mingapi kwenye Uibuaji wa kifua kikuu? [__|__]

Maswali ya wazi:
Uzoefu katika uibuaji wa Kifua kikuu

1. Je, una maoni gani kuhusu ugonjwa wa TB kwa waraibu wa dawa za kulevya?
2. Je, unaelewa nini unaposikia kuhusu uibuaji wa kifua kikuu kwa waraibu wa dawa za kulevya?
3. Kwa nini uibuaji wa kifua kikuu unafanyika kwa waraibu wa dawa za kulevya? Faida
4. Ninataka kuelewa jinsi unavyoenda kutafuta na kuchunguza waraibu wa dawa za kulevya kwa ajili ya mpango wa Uibuaji wa kifua kikuu. Je, unaweza kunipitisha kwenye mchakato wako kuanzia mwanzo hadi mwisho

- Ni zana/nyenzo gani unahitaji kama kijana rika, mfano., zana za uchunguzi, zana za upimaji
    - Je, unaamuaje mahali pa kwenda na nani wa kuchunguza?
    - Nyenzo za uchunguzi
    - Ukusanyaji wa sampuli, uchunguzi na majibu ya vipimo
    - Kuunganishwa kwenye huduma
    - Kuchunguza watu wa karibu?
    - Mzunguko wa shughuli za Uibuaji wa kifua kikuu

5. Je, unatengenezaje uaminifu mpaka uweze kuingia kwenye jamii?
    - Mbinu za kujenga uhusiano, mifano
    - Je, unahisi kukubalika na waraibu wa dawa za kulevya /mhudumu wa afya?

6. Tafadhali unaweza kuniambia ni nini kinafanya iwe vigumu kwako kufanya uibuaji wa Kifua kikuu kwa waraibu wa dawa za kulevya?
    - mteja kukataa au kutokutoa ushirikiano - kwa nini? pamoja na mifano
    - uhamaji wa mara kwa mara wa waraibu wa dawa za kulevya
    - unyanyapaa unaowazunguka waraibu wa dawa za kulevya
    - Hofu ya kukamatwa na police? Kukimbia?
    - Uonevu/ukatili
    - jinsia - ushawishi katika kuwa rika la kike au la kiume? kwa nini, kwa mifano
    - ukosefu wa motisha / malipo

7. Je, unashughulikia vipi matatizo yaliyotajwa katika swali la 6 hapo juu

8. Tafadhali unaweza kuniambia kuhusu kile kinachokusaidia unapofanya Uibuaji wa Kifua kikuu?
    - Msaada wa kifedha
    - Msaada wa kituo
    - Mashirika yanayosaidia jamii
    - Ushawishi wa rika
    - Mabadiliko yaliyopendekezwa Kwa ajil ya kuboresha

9. Kwa kuzingatia mwingiliano wako na mhudumu wa afya na mhudumu wa afya ngazi ya jamii, waraibu wa dawa za kulevya wanachukuliaje utekelezaji wa shughuli za uchunguzi wa kifua kikuu?
    - Ridhaa
    - Hofu
    - Kukataa

10. Je, unaelewa nini kuhusu programu za kubadilishana sindano (NSP) na maoni yako katika kutumia programu hizi katika kupata waraibu wa dawa za kulevya wakati wa uchunguzi na matibabu ya Kifua kikuu?

11. Kama kijana rika wa waraibu wa dawa za kulevya, ulichaguliwa vipi kutekeleza Uibuaji wa kifua kikuu?
    - Uchaguzi wa bahati nasibu, elezea
    - mafunzo yaliyopokelewa - muda, mzunguko, mafunzo ya mwisho
    - Kwako imekaaje kufanya hii kazi?
    - unajisikiaje kuwa karibu na watu wengine wanaotumia Dawa za Kulevya katika jamii?

12. Je, una maoni yoyote ya nyongeza kuhusu mada hii ambayo ungependa kutushirikisha?

Mwisho wa mahojiano

Mshukukuru Mshiriki

Tuberculosis Active Case Finding among People Who Use Drugs (PWUDs)

In-depth interview – People who Use Drugs

(Version 1.2 dated 20 October 2022)

Interview Information:
Name of Interviewer:
Location:
Interviewee code:
Date:

Introductory Remarks:
Thank you for consenting to participate in this interview. I am......................... and I would like to discuss about your views in relation to Tuberculosis (TB) active case finding (ACF) among people who use drugs (PWUDs). The study is being done by the National Institute for Medical Research – Mbeya Medical Research Centre and Ifakara Health Institute in collaboration with the Swiss Tropical and Public Health Institute, Switzerland.

Closed Questions:
Participant Identification
a) Name of health facility - MAT clinic: ____
b) If not an MAT client name of organization or location where participant was accessed ____________
c) Name of district: ____
d) Age (years): ____

e) Sex: ☐ Male ☐ Female
f) Level of education
   i. No education
   ii. Primary education
   iii. Secondary education
   iv. High school
   v. Vocational training
   vi. University
g) Occupation
   i. Employed (specify)
      - Formal
      - Informal
   ii. Unemployed

h) Have you ever injected illicit drugs? ________ (yes / no)
i) At what age (years) did you start using illicit drugs? ______ years
j) Have you ever been treated for TB? ______ (yes / no)
k) If yes, how many times have you been treated for TB _______

Open Questions:
Acceptability of TB ACF
1. What do you think is the burden of TB disease among PWUDs?
   - Why
2. What do you understand by TB active case finding? Please explain
3. Has anyone ever visited you and asked you about TB symptoms without you presenting yourself to the health facility / seeking the health services? (to differentiate between active and passive TB case finding)
   - Who, when, where?

4. Could you please explain your experience when being screened for TB, that is take us through the process from initial contact with screening to the end of the process, with examples
   - Personnel – the way they presented themselves, the team members
   - Location – place, time, duration, privacy
   - Screening and sample collection – tool, type, done on site
   - Feedback / results process
   - Fear of law enforcement – thought they were going to catch me / run away
   - basing on your interactions with HCW, CHW and peer, who are you most comfortable with when being screened for TB?
   - what you liked most, what you did not like and what you would have changed for a better experience?

5. What is your understanding on needle syringe exchange programs (NSP) and your opinion in using these programs in accessing PWUDs during TB screening and treatment?

6. Would you be interested in becoming a peer in TB ACF? Please explain

7. Do you have any additional comments on this topic that you would like to share?

End of interview

Thank the participant/respondent

Uibuaji wa Kifua Kikuu kwa waraibu wa dawa za kulevya (PWUDs)

Mahojiano ya Kina – Waraibu wa dawa za kulevya

(Toleo la 1.2 tarehe 20 Octoba 2022)

<u>Taarifa ya Mahojiano:</u>
Jina la anaehoji:
Eneo:
Nambari ya anaehojiwa:
Tarehe:

Taarifa za Utangulizi:
Asante Kwa kukubali kushiriki kwenye mahojiano haya. Mimi ni ....na ningependa tujadili kuhusu maoni yako kuhusuiana na Uibuaji wa Kifua kikuu kwenye jamii ya waraibu wa dawa za kulevya. Utafiti unafanywa na Taasisi ya Taifa ya Utafiti wa Magonjwa ya Binadamu - Kituo Cha Utafiti Mbeya na Taasisi ya Afya Ifakara Kwa kushirikiana na Taasisi ya Afya ya Jamii ya Swiss Tropical, Uswisi.

<u>Maswali yaliyofungwa:</u>
<u>Utambulisho wa Mshiriki</u>

a) Jina la kituo cha afya – Kliniki ya MAT____
b) Kama si mteja wa MAT kliniki, jina la taasisi au eneo ambalo mshiriki alihojiwa__________________
c) Jina la Wilaya: ____
d) Umri (miaka): ____

e) Jinsia: ☐ Me ☐ Ke
f) Kiwango Cha elimu
   i. Hajasoma
   ii. Elimu ya msingi
   iii. Elimu ya sekondari
   iv. Elimu ya juu
   v. Mafunzo ya ufundi
   vi. Chuo kikuu
g) Kazi yake
   i. Umeajiriwa (taja)
      - Rasmi
      - Isiyo rasmi
   ii. Hajaajiriwa

h) Umewahi kujidunga dawa za kulevya? ________ (ndio / hapana)
i) Ulianza kujidunga dawa za kulevya ukiwa na miaka mingapi? ______ miaka
j) Umewahi kutibiwa ugonjwa wa kifua kikuu? ______ (ndio / hapana)
k) Kama ndiyo, ni mara ngapi umetibiwa kifua kikuu? _______

Maswali ya wazi:
Kukubalika Kwa Uibuaji wa Kifua Kikuu

1. Je, unafikiri kuna mzigo gani wa ugonjwa wa kifua kikuu kwa waraibu wa dawa za kulevya?
   - Kwa Nini?
2. Je, unaelewa nini kuhusu Uibuaji wa Kifua Kikuu kwenye jamii? Tafadhali eleza
3. Je, kuna mtu yeyote amewahi kukutembelea na kukuuliza kuhusu dalili za TB bila wewe kujipeleka kwenye kituo cha afya/kutafuta huduma za afya? (kutofautisha kati ya Uibuaji kifua kikuu na kutafuta uchunguzi wa Kifua kikuu mwenyewe)
   - Nani, lini, wapi?

4. Tafadhali unaweza kuelezea uzoefu wako wakati wa kuchunguzwa Kifua Kikuu, namaanisha kutupitisha kwenye mchakato kuanzia mawasiliano ya awali na uchunguzi hadi mwisho wa mchakato, kwa kutoa mifano
    - Wafanyakazi - jinsi walivyojionyesha, washiriki wa timu
    - Mahali - eneo, wakati, muda, faragha
    - Uchunguzi na ukusanyaji wa sampuli - nyenzo, aina, imefanyika kwenye kituo
    - Mchakato wa Majibu/matokeo
    - Hofu ya utekelezaji wa sheria - kufikiria kwamba watanikamata / kukimbia
    - kulingana na uhusiano wako na wahudumu wa afya, wahudumu wa afya ngazi ya jamii na kundi rika, ni nani uko huru naye zaidi unapochunguzwa?
    - ni nini ulipenda zaidi, nini haukupenda na ni nini ungebadilisha ilibiwe bora zaidi?

5. Je, unaelewa nini kuhusu progamu za kubadilishana sindano (NSP) na maoni yako katika kutumia programu hizi kwenye kutathmini watu wanaotumia Dawa za Kulevya wakati wa uchunguzi na matibabu ya TB?

6. Je, ungependa kuwa kijana rika katika Uibuaji wa kifua kikuu? Tafadhali eleza

7. Je, una maoni yoyote ya nyongeza kuhusu mada hii ambayo ungependa kutushirikisha?

Mwisho wa Mahojiano

Mshukukuru Mshiriki
