# Supplementary figures and images for: Facilitators, barriers, and strategies for the implementation of peer-led tuberculosis active case finding among people who use drugs in Dar es Salaam, Tanzania
Source: PLoS One. 2025 May 28;20(5):e0310069. doi: 10.1371/journal.pone.0310069 (PMC12118968; doi:10.1371/journal.pone.0310069)

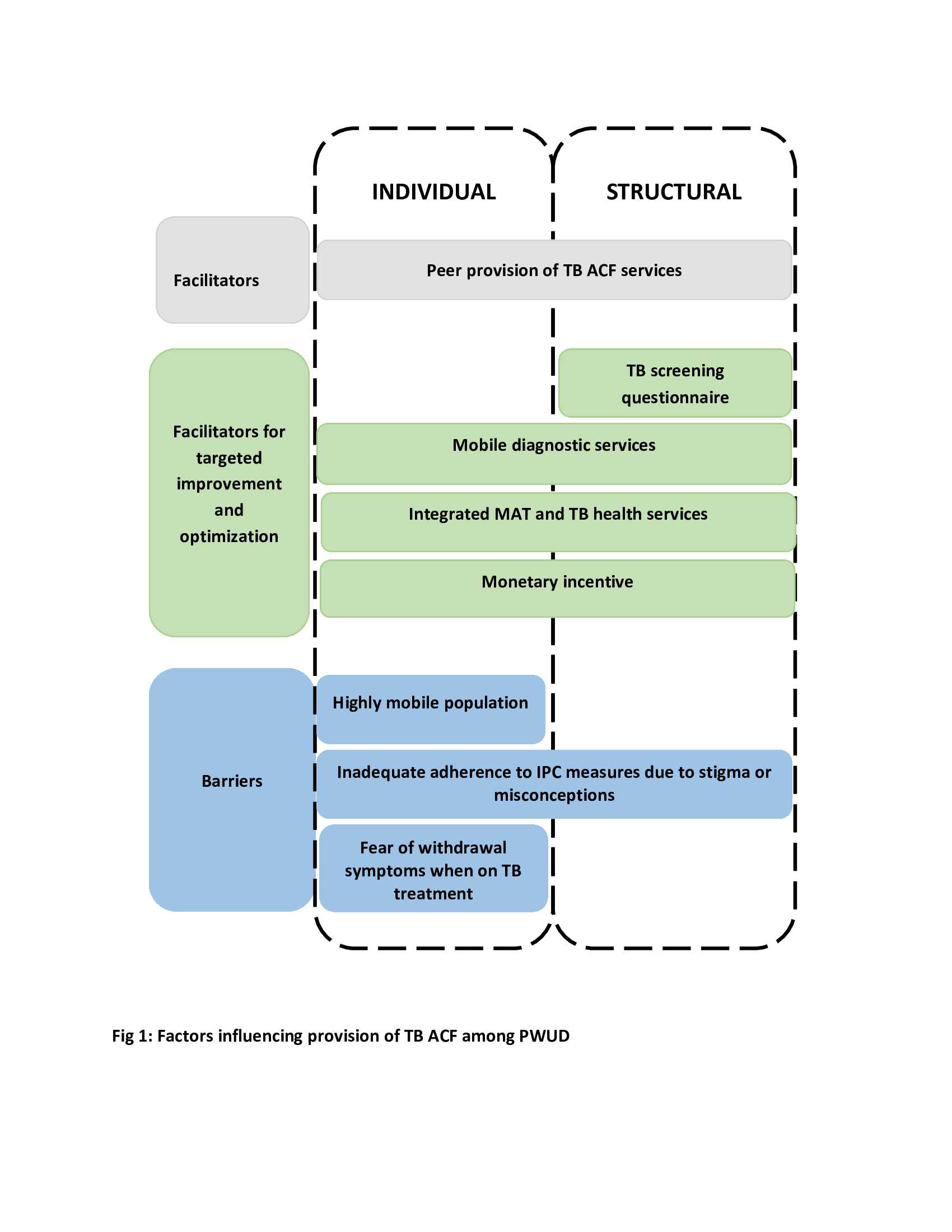

Supplement: S3 Fig — (TIF) [file pone.0310069.s003.tif]
