# Supplementary material for: Facilitators, barriers, and strategies for the implementation of peer-led tuberculosis active case finding among people who use drugs in Dar es Salaam, Tanzania
Source: PLoS One. 2025 May 28;20(5):e0310069. doi: 10.1371/journal.pone.0310069 (PMC12118968; doi:10.1371/journal.pone.0310069)
Supplement: Attachment — Submitted filename: Reviewers comments.docx [file pone.0310069.s004.docx]

The study entitled “Facilitators, barriers, and strategies for the implementation of peer-led tuberculosis active case finding among people who use drugs in Dar es Salaam, Tanzania” is a study that investigates an area that has not been extensively researched in Tanzania. However, some issues need clarification and improvements as detailed in the following sections:

**Title:** Well written

**Abstract:** well written

**Introduction:** Line 62-63: Citation is missing in this sentence.

**Methods:**

- Lines 102-104 (page 5), what are these three districts from which the participants were recruited? It is better to specify for clarity.
- Lines 108-112 (page 5) is not in its appropriate section. It should be included in a separate section for ethics approval. This section (of ethical approval) describes exactly how the informed consent was performed and obtained.
- Line 113 of the Checklist is not well filled out, as the authors claim. When counterchecked with the manuscript, the analysis section misses a lot of information.
- Lines 136-138: This sentence is odd as the qualitative interview is flexible and the tool may be modified during the interview to fit the merging themes. I suggest to be revised
- Line 146: Refusing to be recorded for a participant does not justify exclusion. You could have interviewed without recording and get useful information for your study. This sentence needs to be deleted or provide a good reason for such an abnormal exclusion.
- Line 156: How and who translated the data?
- Lines 162-166: This section needs extensive descriptions to understand how were the data analyzed (how were codes formed? how reliable were the codes? how did you arrive at the themes? Is there any supportive evidence of rigor in the analysis? This information is important as it informs the true findings in the results section.

**Results:**

- Lines 178: I suggest the facilitators be described separately from barriers. E.g. individual and social facilitators. This will improve the clarity of the findings.
- Line 295: Is this a facilitator or barrier? This is not clear. While counterchecking with Fig. 1, I found that this subtheme is written as an inadequate TB Screening Tool. This is not support what is written in narratives and the figure. Please correct this

**Discussion:**

Should also be structured in terms of individual and social facilitators discussed separately from barriers and then discussed logically and sequentially as appears in the results

**Conclusion**

What is the conclusion? The conclusion is missing the author only provided a recommendation.
