# Supplementary material for: Facilitators, barriers, and strategies for the implementation of peer-led tuberculosis active case finding among people who use drugs in Dar es Salaam, Tanzania
Source: PLoS One. 2025 May 28;20(5):e0310069. doi: 10.1371/journal.pone.0310069 (PMC12118968; doi:10.1371/journal.pone.0310069)
Supplement: Attachment — Submitted filename: Response to Reviewers.docx [file pone.0310069.s006.docx]

Journal Requirements:

When submitting your revision, we need you to address these additional requirements.

1. Please ensure that your manuscript meets PLOS ONE's style requirements, including those for file naming. The PLOS ONE style templates can be found at 
<https://journals.plos.org/plosone/s/file?id=wjVg/PLOSOne_formatting_sample_main_body.pdf> and 
<https://journals.plos.org/plosone/s/file?id=ba62/PLOSOne_formatting_sample_title_authors_affiliations.pdf>

**Response:**

Thank you, we will ensure our manuscript meets PLOS ONE’s style requirements.

2. Please include your full ethics statement in the ‘Methods’ section of your manuscript file. In your statement, please include the full name of the IRB or ethics committee who approved or waived your study, as well as whether or not you obtained informed written or verbal consent. If consent was waived for your study, please include this information in your statement as well.

**Response:**

Thank you for your comment. We have included the full name of the IRB and ethics committee that approved the study as well as information on obtaining informed consent. We include the below information on Page 10 and 11, under the ethical considerations section in the revised manuscript:

The study received approvals from the National Health Research Ethics Review Committee and Ifakara Health Institute Institutional Review Board in Tanzania. The investigator welcomed the participant or legally acceptable representative (for illiterate participants) to participate. The investigator then provided the participant with an informed consent form, that provided information on the study, including its potential benefits and risks. Adequate time was provided to the participant to read and ask questions about the study and an assessment of understanding was conducted by the investigator to confirm the participant had understood the information provided. The language used was as non-technical as possible. Written informed consent was obtained from all study participants. In the case of illiteracy, informed oral consent was attested by an impartial witness and documented with the participant’s fingerprint.

3. We note that you have indicated that there are restrictions to data sharing for this study. For studies involving human research participant data or other sensitive data, we encourage authors to share de-identified or anonymized data. However, when data cannot be publicly shared for ethical reasons, we allow authors to make their data sets available upon request. For information on unacceptable data access restrictions, please see <http://journals.plos.org/plosone/s/data-availability#loc-unacceptable-data-access-restrictions>.

Before we proceed with your manuscript, please address the following prompts:

a) If there are ethical or legal restrictions on sharing a de-identified data set, please explain them in detail (e.g., data contain potentially identifying or sensitive patient information, data are owned by a third-party organization, etc.) and who has imposed them (e.g., a Research Ethics Committee or Institutional Review Board, etc.). Please also provide contact information for a data access committee, ethics committee, or other institutional body to which data requests may be sent.

**Response:**

Thank you for the comment regarding data sharing.

We recognize the importance of making data available for further research and enhance transparency. However, as some of the interview transcripts contain identifiable and sensitive information, making the data publicly available would compromise this confidentiality, and the assurance that we gave the participants that their data would remain confidential.

We now include the following data sharing description in the manuscript on Page27,

Data availability statement: Some interview transcripts include identifiable and sensitive information, and publicly releasing the data could compromise participant confidentiality. To protect participant privacy, the data generated and analyzed during this study is not publicly accessible. The data generated and analyzed can however be made available upon reasonable request, provided that the data sharing agreement is approved by the Medical Research Coordinating Committee in Tanzania. For data availability requests, please contact [info@nimr.or.tz](mailto:info@nimr.or.tz).

b) If there are no restrictions, please upload the minimal anonymized data set necessary to replicate your study findings to a stable, public repository and provide us with the relevant URLs, DOIs, or accession numbers. Please see <http://www.bmj.com/content/340/bmj.c181.long> for guidelines on how to de-identify and prepare clinical data for publication. For a list of recommended repositories, please see <https://journals.plos.org/plosone/s/recommended-repositories>. You also have the option of uploading the data as Supporting Information files, but we would recommend depositing data directly to a data repository if possible.

Please update your Data Availability statement in the submission form accordingly.

**Response:** Thank you. We have provided information on the restrictions on data sharing on Page 27 of the revised manuscript and provide contact address for data requests.

 4. Please remove all personal information, ensure that the data shared are in accordance with participant consent, and re-upload a fully anonymized data set.

Note: spreadsheet columns with personal information must be removed and not hidden as all hidden columns will appear in the published file.

Additional guidance on preparing raw data for publication can be found in our Data Policy (<https://journals.plos.org/plosone/s/data-availability#loc-human-research-participant-data-and-other-sensitive-data>) and in the following article: <http://www.bmj.com/content/340/bmj.c181.long>.

**Response:** Thank you. We have provided information on the restrictions on data sharing on Page 27 of the revised manuscript and provide contact address for data requests.

Reviewers' comments:

Reviewer's Responses to Questions

**Comments to the Author**

1. Is the manuscript technically sound, and do the data support the conclusions?

The manuscript must describe a technically sound piece of scientific research with data that supports the conclusions. Experiments must have been conducted rigorously, with appropriate controls, replication, and sample sizes. The conclusions must be drawn appropriately based on the data presented.

Reviewer #1: Partly

Reviewer #2: Partly

Reviewer #3: Partly

Reviewer #4: Yes

**Response:** Thank you for your valuable and comprehensive review. Following your valuable suggestions and comments, we provide detailed description on how our study was conducted in the revised manuscript, ensuring the rigorous conduct of our study is conveyed clearly in the manuscript. We provide our revised manuscript for your review and consideration for publication.

2. Has the statistical analysis been performed appropriately and rigorously?

Reviewer #1: No

Reviewer #2: Yes

Reviewer #3: No

Reviewer #4: I Don't Know

**Response:** Thank you for your comprehensive review. We have addressed comments and suggestions provided on the study analysis. Page 9 and 10 of our revised manuscript provides detailed information on how our study analysis was conducted reflecting the appropriate and rigorous aspects that were done. The data analysis section in the revised manuscript reads:

Data analysis: Thematic analysis was used to analyze the qualitative data using NVivo 12 software. The audio recordings were transcribed verbatim in Swahili before translation into English. The data was translated by a professional translator fluent in both the local language Swahili and English. The translator was familiar with the context of the study to ensure accuracy and cultural relevance. The translated transcripts and field notes were further double checked for accuracy by the research team (LTM, DP, KM) thus also allowing for familiarization of data. In order to develop initial draft codebooks to guide subsequent coding, LTM, DP and KM randomly selected one transcript from each of the three participant groups thus, each with a transcript to work on. Deductive codes were applied based on research questions and few others were emergent from the transcripts. Thereafter, the team met for a group review of the coded transcripts. During the review, the team extensively reviewed the codebooks. When discrepancies arose, agreements were reached through discussions. Thus, certain codes were deleted, merged and rephrased. The final codebooks were subsequently applied to the rest of the transcripts which were randomly divided among the team. Thereafter, codes were grouped into categories and themes were developed from categories with similar meaning.

3. Have the authors made all data underlying the findings in their manuscript fully available?

The [PLOS Data policy](http://track.editorialmanager.com/CL0/http:%2F%2Fwww.plosone.org%2Fstatic%2Fpolicies.action%23sharing/1/010f019481d90c86-0c1e6650-9ac2-4282-88d6-b1c131e12c78-000000/T_ZGZhXZH5zlvnpBoiwve6uAIAmz5JeqP86-lHlZWuM=194) requires authors to make all data underlying the findings described in their manuscript fully available without restriction, with rare exception (please refer to the Data Availability Statement in the manuscript PDF file). The data should be provided as part of the manuscript or its supporting information, or deposited to a public repository. For example, in addition to summary statistics, the data points behind means, medians and variance measures should be available. If there are restrictions on publicly sharing data—e.g. participant privacy or use of data from a third party—those must be specified.

Reviewer #1: Yes

Reviewer #2: No

Reviewer #3: No

Reviewer #4: Yes

**Response:**

Thank you for the detailed review on data sharing.

We recognize the importance of making data available for further research and enhance transparency. However, as some of the interview transcripts contain identifiable and sensitive information, making the data publicly available would compromise this confidentiality and the assurance that we gave the participants that their data would remain confidential.

We now include the following data sharing description in the manuscript on Page 27

Data availability statement: Some interview transcripts include identifiable and sensitive information, and publicly releasing the data could compromise participant confidentiality. To protect participant privacy, the data generated and analyzed during this study is not publicly accessible. The data generated and analyzed can however be made available upon reasonable request, provided that the data sharing agreement is approved by the Medical Research coordinating Committee in Tanzania. For data availability requests, please contact [info@nimr.or.tz](mailto:info@nimr.or.tz)

4. Is the manuscript presented in an intelligible fashion and written in standard English?

PLOS ONE does not copyedit accepted manuscripts, so the language in submitted articles must be clear, correct, and unambiguous. Any typographical or grammatical errors should be corrected at revision, so please note any specific errors here.

Reviewer #1: Yes

Reviewer #2: Yes

Reviewer #3: Yes

Reviewer #4: Yes

**Response:**

Thank you for the comment, we appreciate your feedback.

5. Review Comments to the Author

Please use the space provided to explain your answers to the questions above. You may also include additional comments for the author, including concerns about dual publication, research ethics, or publication ethics. (Please upload your review as an attachment if it exceeds 20,000 characters)

Reviewer #1: The study entitled “Facilitators, barriers, and strategies for the implementation of peer-led tuberculosis active case finding among people who use drugs in Dar es Salaam, Tanzania” is a study that investigates an area that has not been extensively researched in Tanzania. However, some issues need clarification and improvements as detailed in the following sections:

Title: Well written

Abstract: well written

**Response:** Thank you for the positive feedback on the title and abstract.

Introduction: Line 62-63: Citation is missing in this sentence.

**Response:** Thank you for noting this and we apologize for missing to add the citation.

In our revised manuscript, we have added the below citation (Citation number 11 of the revised manuscript) to the sentence to ensure proper referencing and compliance with the guidelines.

*WHO consolidated guidelines on tuberculosis systematic screening for tuberculosis disease; Module 2: Screening. Geneva, Switzerland: World Health Organization; 2021.*

Methods:

• Lines 102-104 (page 5), what are these three districts from which the participants were recruited? It is better to specify for clarity.

**Response:**

Thank you for your suggestion.

We have specified the three districts from which the participants were recruited on Page 6 of the revised manuscript as shown below:

Participants were recruited from three different MAT clinics located in Ilala, Kinondoni and Temeke districts.

• Lines 108-112 (page 5) is not in its appropriate section. It should be included in a separate section for ethics approval. This section (of ethical approval) describes exactly how the informed consent was performed and obtained.

**Response:**

We agree that the information regarding informed consent would be more appropriately placed under the ethics approval section. We have moved the content to the relevant section under Ethical considerations on page 10 and 11 of the revised manuscript.

• Line 113 of the Checklist is not well filled out, as the authors claim. When counterchecked with the manuscript, the analysis section misses a lot of information.

**Response:** We apologize for the oversight in the COREQ checklist. We have revised the analysis section in the manuscript and provide detailed information to ensure that all relevant information is included and accurately reflects the content. An updated COREQ checklist has been appended.

The data analysis section (Page 9 and 10) in the revised manuscript reads:

Data analysis: Thematic analysis was used to analyze the qualitative data using NVivo 12 software. The audio recordings were transcribed verbatim in Swahili before translation into English. The data was translated by a professional translator fluent in both the local language Swahili and English. The translator was familiar with the context of the study to ensure accuracy and cultural relevance. The translated transcripts and field notes were further double checked for accuracy by the research team (LTM, DP, KM) thus also allowing for familiarization of data. In order to develop initial draft codebooks to guide subsequent coding, LTM, DP and KM randomly selected one transcript from each of the three participant groups thus, each with a transcript to work on. Deductive codes were applied based on research questions and few others were emergent from the transcripts. Thereafter, the team met for a group review of the coded transcripts. During the review, the team extensively reviewed the codebooks. When discrepancies arose, agreements were reached through discussions. Thus, certain codes were deleted, merged and rephrased. The final codebooks were subsequently applied to the rest of the transcripts which were randomly divided among the team. Thereafter, codes were grouped into categories and themes were developed from categories with similar meaning.

• Lines 136-138: This sentence is odd as the qualitative interview is flexible and the tool may be modified during the interview to fit the merging themes. I suggest to be revised

**Response:** As you correctly pointed out, qualitative interview guides are inherently flexible, and this was carefully considered during the study. We have revised the section on Page 8 and as shown below to clarify how the interviews were conducted, emphasizing the flexibility of the interview process to accommodate emerging themes.

The interview guides were piloted on two peer PWUD and one MAT client. Feedback from pilot interviews indicated that the questions were well understood and no substantive changes were needed in the interview guides. However, during subsequent interviews, questions in the interview guides were rephrased or added depending on the participants responses and probing skills.

• Line 146: Refusing to be recorded for a participant does not justify exclusion. You could have interviewed without recording and get useful information for your study. This sentence needs to be deleted or provide a good reason for such an abnormal exclusion.

**Response:** As you correctly highlighted, interview notes could have been an alternative to recording. However, for consistency in the level of detail that could be extracted from participants’ interviews, we wanted to ensure that all interviews could be captured verbatim, requiring audio recording. For this reason, it was included as a study inclusion criterion, which was approved by the ethics board and stipulated in the informed consent form. The decision not to proceed when a participant refuses to be audio recorded was based on the approved informed consent form.

We have clarified this on Page 8 and 9 of the manuscript to provide a better explanation for the exclusion. We have taken note of this and will consider incorporating this approach in future studies.

• Line 156: How and who translated the data?

**Response:** Thank you.

We have updated the manuscript for added clarity as shown below and on Page 9 and 10 of the manuscript;

The data was translated by a professional translator fluent in both the local language Swahili and English. The translator was familiar with the context of the study to ensure accuracy and cultural relevance. The translated transcripts and field notes were further double checked for accuracy by the research team (LTM, DP, KM) thus also allowing for familiarization of data.

• Lines 162-166: This section needs extensive descriptions to understand how were the data analyzed (how were codes formed? how reliable were the codes? how did you arrive at the themes? Is there any supportive evidence of rigor in the analysis? This information is important as it informs the true findings in the results section.

**Response:** Thank you for your comment. We understand the importance of providing clear and detailed explanation of the data analysis process. We have revised the section to include a comprehensive description of how the data were analyzed, including how codes were formed, reliability of codes and the process of identifying themes, as shown below and on Page 9 and 10 of the manuscript;

Data analysis: Thematic analysis was used to analyze the qualitative data using NVivo 12 software. The audio recordings were transcribed verbatim in Swahili before translation into English. The data was translated by a professional translator fluent in both the local language Swahili and English. The translator was familiar with the context of the study to ensure accuracy and cultural relevance. The translated transcripts and field notes were further double checked for accuracy by the research team (LTM, DP, KM) thus also allowing for familiarization of data. In order to develop initial draft codebooks to guide subsequent coding, LTM, DP and KM randomly selected one transcript from each of the three participant groups thus, each with a transcript to work on. Deductive codes were applied based on research questions and few others were emergent from the transcripts. Thereafter, the team met for a group review of the coded transcripts. During the review, the team extensively reviewed the codebooks. When discrepancies arose, agreements were reached through discussions. Thus, certain codes were deleted, merged and rephrased. The final codebooks were subsequently applied to the rest of the transcripts which were randomly divided among the team. Thereafter, codes were grouped into categories and themes were developed from categories with similar meaning.

Results:

• Lines 178: I suggest the facilitators be described separately from barriers. E.g. individual and social facilitators. This will improve the clarity of the findings.

**Response:** Thank you for your suggestion. We agree that separating facilitators from barriers, and categorizing them into individual and or social will enhance the clarity of the findings.

We have reorganized the section accordingly, ensuring that facilitators and barriers are described separately. Additionally, we have added a new theme, "Facilitators for targeted improvement and optimization," to better reflect the facilitators that needed to be optimized. This updated structure enhances the clarity and flow of the findings.

• Line 295: Is this a facilitator or barrier? This is not clear. While counterchecking with Fig. 1, I found that this subtheme is written as an inadequate TB Screening Tool. This is not support what is written in narratives and the figure. Please correct this

**Response:** Thank you for pointing this out and we appreciate your careful review.

The TB screening tool was, in fact, a facilitator for targeted improvement and optimization, rather than a barrier. We have revised the section to better reflect this and have added a new theme to clarify its role as a facilitator. Additionally, we have updated the manuscript as well as Fig. 1 to ensure consistency between the narrative and the figure. We provide the below figure in the revised manuscript

Discussion:

Should also be structured in terms of individual and social facilitators discussed separately from barriers and then discussed logically and sequentially as appears in the results

**Response:** Thank you for your valuable feedback.

We agree that discussing the facilitators separate from barriers, will improve clarity. We have reorganized the section accordingly, ensuring that facilitators and barriers are discussed logically and sequentially as they appear in the results. Additionally, we have added a new theme, "Facilitators for Targeted Improvement and Optimization," to better reflect the facilitators that needed to be optimized. This updated structure enhances the clarity and flow of the findings.

Conclusion

What is the conclusion? The conclusion is missing the author only provided a recommendation.

**Response:** Thank you for your feedback

We have added a conclusion as shown below to the revised manuscript to clearly summarize our findings. The conclusion now highlights the crucial role of peer-led approaches in enhancing TB active case finding (ACF) among people who use drugs (PWUD). It emphasizes the potential of community-driven interventions, particularly peer acceptance as service providers. We also discuss the importance of strengthening facilitators and addressing challenges to optimize these services. Additionally, we recommend that future research explore the feasibility of peer-supported TB diagnosis and treatment at accessible, welcoming drop-in centers.

Conclusion: Our findings highlight the crucial role of peer-led approaches in enhancing TB ACF among PWUD. Peer acceptance as service providers highlights the potential of community-driven interventions. Strengthening facilitators and addressing challenges is key to optimizing these services.

Reviewer #2: Interesting article focusing on an important population. Given the importance of HIV as the number one risk factor for TB in Tanzania (estimated 20,000 are coinfected) and the increased rates of HIV in PWUD the lack of mentioning of HIV throughout the article needs addressed - a 2017 IBBS survey estimated the overall prevalence of HIV infection in PWUD in Tanzania was 8.7% (95% CI 6.5–10.9). Several studies conducted in Tanzania between 2010 and 2018 have reported that the prevalence of HIV infection among PWID ranges between 11.0 and 51.1% (Likindikoki, S.L., Mmbaga, E.J., Leyna, G.H. et al. Prevalence and risk factors associated with HIV-1 infection among people who inject drugs in Dar es Salaam, Tanzania: a sign of successful intervention? Harm Reduct J 17, 18 (2020). From reviewing the questionnaires, for the Peer PWUD the HUV status was requested but not for the PWUD, which was a missed opportunity given the importance of this as a risk factor.

For the introduction section:

- please use the latest numbers from WHO Global TB report 2024, for example WHO Global TB report 2024 reports the incidence is now 183 per 100,000 for Tanzania.

- Can more information be given on the situation regarding PWUD in Tanzania; any updated IBBS or other data on rates of coinfection with HIV/HCV, what is the estimated burden of PWUD in Tanzania? Any data on the number of people who inject drugs, what is the legal situation regarding drug use in Tanzania (this may impact discussions around stigmatization and accessing care)

**Response:** Thank you for your comment.

We agree that HIV is a critical risk factor for TB, particularly in the context of people who use drugs (PWUD) in Tanzania. We have revised the manuscript to highlight the role of HIV as well as other key risk factors for TB in this population. Unfortunately, the most recent data on PWUD prevalence in Tanzania dates back to 2014. However, we have included available 2017 IBBS data on HIV/HCV co-infection to provide a more comprehensive picture of the health challenges faced by this population. Additionally, we state the below in the revised manuscript on Page 5 and 6

An integrated bio-behavioral survey (IBBS) conducted among PWID in Dar es Salaam, Tanzania in 2017 reports an HIV prevalence of 8.7% and an HIV/HCV co-infection prevalence at 43.1% (16,20). The prevalence of 8.7% among PWID is a notable decrease from 15.5% in 2015 (17), however, this figure is nearly double the national HIV prevalence of 4.4% (24).

From reviewing the questionnaires, for the Peer PWUD the HUV status was requested but not for the PWUD, which was a missed opportunity given the importance of this as a risk factor.

**Response:**

Thank you for this comment.

While we had information on HIV status for the peer PWUD and MAT clients through the MAT clinics (Page 11), we unfortunately missed this data, among community PWUD. We have addressed the gap in obtaining the HIV status among community PWUD in the limitations section and suggest that future studies consider mixed method research to enable in depth capture of information

- With regards to current community active case finding activities more generally, can sputum samples be collected in the community or do people have to attend a health care facility? This knowledge can help understand the recommendation regarding access to diagnostic services/strengthening peer ACF if they can collect samples in the community.

**Response:** Thank you for highlighting this important aspect.

When conducting enhanced TB Active Case Finding (ACF), sputum samples can indeed be collected directly within the community and transported to the diagnostic facility by the community health worker or peer involved in TB ACF. Alternatively, the TB ACF provider may accompany or refer the client to the mobile diagnostic van for testing or refer the client to a nearby diagnostic health facility. This information is specified on page 5 of the revised manuscript.

For the Recruitment and sample selection:

- Was any consideration given to HIV status in the sample selection given prevalence and risk factor for TB?

**Response:** We appreciate this feedback and have updated the manuscript to reflect the inclusion and results of HIV status as a factor in the sample selection process among the peer PWUD and MAT clients. We did not collect the HIV from the community PWUD, which represents a missed opportunity. We have added this as a limitation in the updated manuscript and suggest that future studies ensure comprehensive HIV data collection across all subgroups of PWUD, including community PWUD. This information is found on page 11, with limitations on page 24.

- More information regarding the Peer PWUD would be helpful, such as what other peer activities do they perform (if any), were all of them involved in TB ACF? Reading later in the article it would seem that is the case but would be good to have the specifically stated when outlining the groups interviewed? Was any duration of time involved in ACF discussed, given that that was one of the questions asked?

**Response:** We have updated the manuscript to provide more detailed information regarding peer PWUD and their roles. In addition to their involvement in TB ACF, peer PWUD also engage in other harm reduction services such as needle syringe program and HIV services. We have clarified in the manuscript that all the peer PWUD interviewed were involved in TB ACF, as this was crucial to the study. The revised manuscript reads;

All peer PWUD interviewed were experienced in providing TB ACF and were selected from the MAT clinics, based on their good conduct and positive progress at the MAT clinics. Some peer PWUD would additionally be involved in other harm reduction services such needle syringe program and HIV services.

- Was the interview location in a private room? It mentions being in the treatment center but did not say if it was in a more public/open space or private space.

Yes, the interviews were conducted in a private space to ensure confidentiality and comfort for the participants. Specifically, this was a room at the Medication-Assisted Treatment (MAT) clinic or at the community-based organization. This setting was chosen to provide a conducive environment for open and honest discussions. We have updated the manuscript to clarify this point.

**Response:** Thank you for the comment. Interviews were conducted in a private place.

In Page 9 of the revised manuscript the below statement can be read;

Interviews were conducted in a private room at the local MAT clinic or at the partner community-based organization to ensure confidentiality.

Results:

- Were there any perceived issues or reflections regarding the population being interviewed being mainly male and the interviewees mainly female? Were the interviewees given any choice regarding who interviewed them?	Thank you for your thoughtful comment.

**Response:** Thank you for this important comment. Yes, we did consider the gender dynamics in the interviews and have updated the manuscript to include the below information on Page 9 of the manuscript.

After being invited to participate in the study and providing informed consent, participants were asked if they were comfortable and willing to be interviewed by the interviewer, regardless of gender. None of the interviewed participants expressed any gender preferences.

- The previous TB history is important and more information regarding how many of the interviewees had a past history as this may have an impact on their responses regarding TB ACF and starting/staying in treatment, where peer PWUD more likely to have had a previous episode of TB? Did this help their work in engaging with PWUD? This data appears to have been recorded in both groups.

**Response:** Thank you for the comment. Prior history of TB was reported in 41%. However, no obvious predominance was noted among the peer PWUD and MAT client groups as documented on page 11.

- The lack of information on HIV status makes the statements on IPC being identified as a risk factor for multiple episodes of TB when the main risk factor for TB is unknown (HIV). Additional risk factors such as malnutrition are als0 not mentioned. These multiple episodes of TB, was any time frame collected on this? Was this seen in Peer PWUD who had been doing it for a long period of time? What was the experience range of the peer PWUD?

**Response:** We appreciate this feedback and have updated the manuscript to reflect the inclusion of HIV status as a factor in the sample selection process among the peer PWUD and MAT clients. We have also included information on HIV and other TB risk factors in the introduction section of the revised manuscript.

We have updated the revised manuscript to include the HIV status among this purposively selected population. We report on the HIV status of peer PWUD and MAT clients, as we did not collect the HIV status from the community PWUD. However, we believe it is important to include information on IPC measures even in the absence of supportive quantitative data from all groups, as this was a perspective shared by participants. We base this on the fact that qualitative analysis focuses on capturing participants' experiences and insights, and does not draw statistical conclusions.

Regarding multiple episodes of TB, while we gathered information on prior TB history, we did not specifically collect the exact timepoints of when participants developed TB disease.

The experience range of the peer PWUD varied between 1 and 6 years, we have updated the manuscript to include this.

- The discussions around usefulness of the TB screening questions when a positive symptom screen results in a negative test can not be fully understood without knowledge of the HIV status of the population - PLHIV are more likely to be paucibacillary and therefore more likely to have negative bacteriological samples regardless of PWUD status.

**Response:** Thank you for your insightful comment.

We agree that HIV is a critical risk factor for TB, particularly in the context of people who use drugs (PWUD) in Tanzania. The purpose of our study was to explore facilitators and barriers to implementing peer-led TB ACF, as perceived by both current and potential service users, using a qualitative approach to understand participants experiences and insights. Our discussion then centers around the findings about facilitators, facilitators for improvement and optimization and barriers to peer led TB ACF among PWUD. However, we provide prevalence in this purposively selected group of peer PWUD and MAT clients. To support our point, we also draw on literature showing a high prevalence of HIV among PWUD as detailed in the introduction section.

For Line 355 regarding the drop in services - are they already offering any TB screening/diagnostic services? It isn't clear if this is something that needs to start or can be expanded.

**Response:** Thank you for your comment.

To clarify, drop-in centers do not currently offer TB diagnostic services. However, they act as a vital link between community PWUD and diagnostic health facilities. These centers provide essential support and help facilitate access to health services, including TB diagnostics, but the actual diagnostic services are typically provided at nearby health facilities. We have updated the manuscript to reflect this distinction and to clarify the role of drop-in centers in connecting PWUD with the appropriate diagnostic services (Page 22)

Drop-in centers do not currently offer TB diagnostic services, however act as a vital link between community PWUD and health facilities. This approach has the potential to reduce attrition rates in TB services by providing screening, diagnosis, and treatment in one location.

Reviewer #3: A review of the manuscript entitled “Facilitators, barriers, and strategies for the implementation of peer-led tuberculosis active case finding among people who use drugs in Dar es Salaam, Tanzania.”

1. (page 3, lines 58-60): Please use the most updated data on the number of TB cases in the Tanzanian population.

**Response:** Thank you for the comment.

We have updated the information to reflect the most recent data on TB cases in Tanzania, using the latest figures from the WHO Global TB Report 2024 and the country National TB and Leprosy data as shown on Page 4 of the manuscript which reads as shown below

Globally, tuberculosis (TB) is again the leading cause of death from a single infectious agent. In 2023, there were an estimated 2.7 million undiagnosed TB cases worldwide (1). Tanzania is among the 30 high TB burden countries globally, with an estimated TB incidence of 183 cases per 100,000 population

2. (page 3, lines 55-56): This sentence “Globally, tuberculosis (TB) is again the leading cause of death from a single infectious agent” will be more significant if it’s also supported by another relevant study. For example, “Risk factors for viral hepatitis in pulmonary tuberculosis patients undergoing treatment: A systematic review and meta-analysis”.

**Response:** Thank you for your comment.

We agree that supporting this statement and manuscript with additional relevant studies will strengthen the argument. We have now included the referenced study below and in citation number 24 of the revised manuscript.

“Risk factors for viral hepatitis in pulmonary tuberculosis patients undergoing treatment: A systematic review and meta-analysis,” to provide further evidence on co-infections and their impact. This addition enhances the discussion on the burden of TB as the leading cause of death from a single infectious agent. Ilham AF, Andini SR, Afladhia HL, Rakasiwi MID, Burhan E. Risk factors for viral hepatitis in pulmonary tuberculosis patients undergoing treatment: A systematic review and meta-analysis. Narra J. 2024 Dec;4(3):e1242.

3. (page 5, lines 93-95): The motivation of the research is not clearly presented. What are the novelty aspects of this work?

**Response:** Thank you for your comment. We have revised the manuscript to better articulate the motivation and novelty of the study. We have included the below in the revised manuscript on Page 6;

Despite high incidence and prevalence of TB among PWUD, there is a significant gap in data, on their perspectives, regarding implementation of TB ACF services. This study aimed to explore facilitators and barriers to implementing peer-led TB ACF, as perceived by both current and potential service users.

4. (page 5, study setting and design): Why did the authors only recruit participants from three MAT clinics? In my view, a larger sample size will make the study findings stronger and more powerful. The current number of sample size seems somehow small to make the conclusion drawn from this study significant.

**Response:** Thank you for your comment.

Participants were recruited from three MAT clinics, but in addition, PWUD from the community were also included. The three selected MAT clinics included the first clinics to provide methadone in the country. While we acknowledge that a larger sample size could enhance the strength of the study's findings. We followed guidelines for qualitative research sample size, which focus on data saturation and information power. We reached these targets in our data collection, which suggest that a larger sample size would not be necessary. This allowed for meaningful insights to be drawn from the study. We are also aware that the goal of qualitative work is not to produce generalizable findings but rather to understand a specific experience in depth. Toward this end, we ensure that our discussion and conclusions do not overstate the implications of the findings.

5. (page 8, lines 162-166): The data analysis part seems somewhat questionable as the authors did not give detailed information on how the data was analysed.

**Response:** Thank you for your valuable comment.

We understand the importance of providing clear and detailed explanation of the data analysis process. We have revised the section to include a comprehensive description of how the data were analyzed, including how codes were formed, reliability of codes and the process of identifying themes. We have also outlined how discrepancies were resolved through team discussions as shown in the below section in the revised manuscript;

Data analysis: Thematic analysis was used to analyze the qualitative data using NVivo 12 software. The audio recordings were transcribed verbatim in Swahili before translation into English. The data was translated by a professional translator fluent in both the local language Swahili and English. The translator was familiar with the context of the study to ensure accuracy and cultural relevance. The translated transcripts and field notes were further double checked for accuracy by the research team (LTM, DP, KM) thus also allowing for familiarization of data. In order to develop initial draft codebooks to guide subsequent coding, LTM, DP and KM randomly selected one transcript from each of the three participant groups thus, each with a transcript to work on. Deductive codes were applied based on research questions and few others were emergent from the transcripts. Thereafter, the team met for a group review of the coded transcripts. During the review, the team extensively reviewed the codebooks. When discrepancies arose, agreements were reached through discussions. Thus, certain codes were deleted, merged and rephrased. The final codebooks were subsequently applied to the rest of the transcripts which were randomly divided among the team. Thereafter, codes were grouped into categories and themes were developed from categories with similar meaning. (Page 9 and 10)

6. (page 18, lines 373-375): This sentence “Nonetheless, studies from COVID-19 suggest that uptake of wearing face masks can be enhanced by strengthening the belief of solidarity and promotion of positive social messages” also shares the same idea with a similar study by Hamdan et al entitled “Coping strategies used by healthcare professionals during COVID-19 pandemic in Dubai: A descriptive cross-sectional study”. Kindly include this reference to make the sentence stronger.

**Response:** Thank you for this suggestion. We have included the below reference to make the sentence stronger. Citation number 46 in the revised manuscript

Hamdan A, Eastaugh J, Snygg J, Naidu J, Alhaj I. Coping strategies used by healthcare professionals during COVID-19 pandemic in Dubai: A descriptive cross-sectional study. 2023 Apr 30

7. (page 19, lines 392-399): The last paragraph of the discussion part should be rewritten, especially in terms of highlighting the limitations of your study. The current wording is incorrect because the authors mentioned that this study has numerous strengths, and then in the elaboration they included the study’s weaknesses, which does not flow well with the previous statement of study strengths. Please revise.

**Response:** Thank you for your helpful feedback.

We understand the need for better clarity and flow between the strengths and limitations of the study. We have revised the paragraph on Page 24 of the discussion to ensure more clarity as shown below;

This study has numerous strengths, including the ability to gather insights from three different groups of PWUD: that is those in the community, those attending MAT clinics on methadone, and PWUD who have been successfully weaned off illicit drugs. This allowed for a broader understanding of their experiences and perspectives. In addition, partnering with the country’s first three MAT clinics provided an opportunity to engage with experienced and clients newly enrolled into MAT clinics. This enabled obtaining practical, real-world information on the topic.

The study has several limitations. First, it was conducted in a single region, which may limit the generalizability of the findings to other parts of the country. Second, the HIV status of community PWUD, and the timelines of previous TB episodes were not captured in detail, representing a missed opportunity to further enrich our understanding of TB among PWUD. To address these limitations, we recommend that future studies consider incorporating comprehensive data on TB risk factors, better captured through mixed method research.

Reviewer #4: The manuscript is well-written, and depicts the need to enhance TB services for PWUD to provide indiscriminate care and support. The discussion part is well-versed and highlights the ways forward. Although mobile TB van services are available, it is sad to know it only runs a couple times in a year, indicating the need to improve the integrated services. Overall, an informative and much needed manuscript for this specific population.

Response: Thank you for your thoughtful and positive feedback.

We truly appreciate your recognition of the manuscript's focus on enhancing TB services for PWUD and the need for indiscriminate care and support. We also value your comments on the availability and frequency of mobile TB van services. We agree that increasing the availability of these services is crucial for improving access to TB diagnostics and care for PWUD.
